# ELABELA: a polypeptide with therapeutic potential in cardiovascular diseases

**DOI:** 10.3389/fcvm.2025.1623215

**Published:** 2025-09-01

**Authors:** Ping Yan, Cuicui Meng, Shujun Yang, Weiqing Huang, Tong Wang

**Affiliations:** ^1^ Department of General Medicine, The First Affiliated Hospital of Guangzhou Medical University, Guangzhou, Guangdong, China; ^2^ Department of Emergency, The Eighth Affiliated Hospital of Sun Yat-sen University, Shenzhen, Guangdong, China; ^3^ Department of Emergency, The First Affiliated Hospital of Guangzhou Medical University, Guangzhou, Guangdong, China

**Keywords:** ELA, APJ, cardiovascular disease, diagnostic, therapeutic target

## Abstract

Cardiovascular diseases (CVDs) are an increasingly common public health problem, and their diagnosis and treatment continue to face serious challenges, making it necessary to explore new therapeutic approaches. The small molecule polypeptide ELABELA (ELA) plays an important role in CVDs. We aim to review the role of ELA in cardiovascular development and its related mechanisms, as well as its diagnostic value and therapeutic potential in CVDs, including preeclampsia, hypertension, vascular calcification, various heart diseases, and ischemia–reperfusion injury. ELA can be used as a diagnostic marker for CVDs and has been demonstrated to reduce oxidative stress, cell apoptosis, tissue fibrosis, and vascular hypertension and promote angiogenesis. This suggests that ELA may serve as both a diagnostic and treatment target. This review provides some strategies for the diagnosis and treatment of small molecule polypeptide ELA in CVDs and the development of targeted drugs.

## 1 Introduction

### 1.1 The current status of cardiovascular diseases

Cardiovascular diseases (CVDs) represent an increasingly common public health problem and remain the leading cause of death worldwide. In 2017, CVDs accounted for 17.9 million deaths globally, marking a 21% increase compared with the previous decade (1). CVDs primarily encompass heart- and vascular system-related diseases, such as hypertension, heart disease, and atherosclerosis, with obesity and poor living habits as major contributing factors (2). At present, the main treatment for CVDs focuses on alleviating symptoms. However, existing drug interventions for CVDs have not reached satisfactory therapeutic outcomes. Therefore, effective interventions are urgently needed, and the development of new drugs remains the direction of unremitting efforts (3).

### 1.2 The discovery of ELABELA/APJ

**Figure 1:**
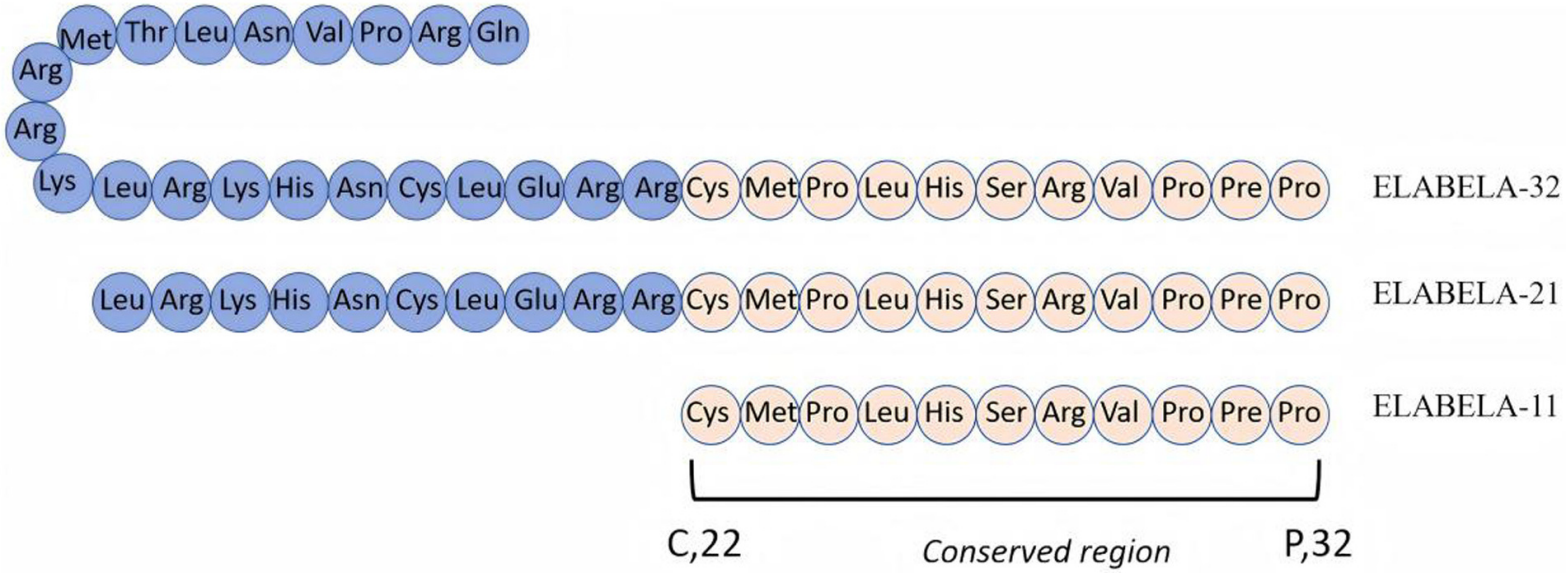
Amino acid sequence of polypeptide ELABELA. Elabela is mainly decomposed by the Golgi apparatus into Elabela-32, Elabela-21, and Elabela-11. Elabela-11 is its conservative area.

Since the advent of insulin in 1992, more than 80 peptide drugs have been used to treat various diseases, and small molecule polypeptides have been developed successively (4). In 2004, Miura et al. (5) discovered the human gene AK092578 through whole-genome sequencing. In 2013, Chng et al. found a conserved reading framework on chromosome 1 in zebra fish embryos, which is processed and cleaved by enzymes in the Golgi apparatus to form Elabela-54, Elabela-32, and Elabela-21 and cleaved by the flynn protease to form the evolutionally conserved 11-peptide Elabela-11 (Figure 1) (6, 7). Elabela-32, also known as Elabela (ELA), is the second new endogenous ligand of the apelin–angiotensin receptor-like receptor (APJ), also known as apela or toddler (8). Prior to this, apelin was the only endogenous peptide binding APJ first discovered in 1998 when it was extracted from bovine stomach tissue (9). The expression time of APJ was different from that of apelin. APJ is expressed before archenteric duct formation, whereas apelin is expressed during the middle and late stages of archenteric duct formation (10). The apelin receptor (APJ, APLNR, AGTRL1) is a seven-transmembrane G-protein-coupled receptor discovered in 1993 due to its sequence similarity to the angiotensin type 1 receptor (AT1). It is located on chromosome 11q12 and is 54% similar to AT1; however, the APJ ligand does not bind to AT1 and is called an “orphan receptor,” indicating a different function than angiotensin II (11). The APJ-deficient mice are born with cardiovascular developmental disorders, while the apelin-deficient mice are virtually unaffected, suggesting the presence of other undiscovered APJ ligands—until 2013, when a second ligand, called ELA, was discovered in zebra fish embryos (6, 12).

ELA is expressed in pluripotent stem cells, embryonic stem cells, kidney and vascular endothelial cells, and coronary arteries (13). Apelin/ELA, APJ, and their downstream signal molecules form the apelinergic system, which plays a series of biological roles. At present, research has found that the apelinergic system plays an important role in many aspects, including embryonic development, cardiovascular and cerebrovascular diseases, and cancer progression (14, 15). However, its mechanisms are still being explored. The purpose of this review is to summarize the role of ELA in cardiovascular pathophysiology and provide some insights into its potential in the diagnosis and treatment of CVDs.

## 2 The role of ELA in embryonic development

### 2.1 ELA promotes embryonic cardiovascular system development

Early embryonic cardiac development relies on the directed differentiation of stem cells (16). Embryonic stem cells can differentiate into the three germ layers, with the mesoderm layer developing into circulatory system components, among others. ELA plays an important role in this differentiation process, and this effect exists independently of the APJ receptor and is mainly achieved by activating the PI3K/Akt pathway (17, 18). In the differentiation stage, ELA upregulates the expression of the cardiac-restricted transcription factors, Tbx5 and GATA4 (19). Loss of apela in mice causes low-penetrance embryonic lethality and defects in early mesodermal derivatives (20). ELA can enhance the transforming growth factor-β (TGF-β) pathway, guiding human embryonic stem cells (hESC) to the endoderm, and is an endogenous growth factor in embryos. In zebra fish embryos, the ELA-APJ system regulates the chemokine (C-X-C motif) receptor 4a (Cxcr4a) molecule between mesoderm and endoderm cells, indirectly promoting endoderm cell migration. ELA knockout can damage cell motility and endoderm differentiation during protoenteroderm formation (21), and endoderm differentiation is very important for the movement of cardiac progenocytes to the cardiac formation area (22). Helker et al. (23) confirmed that ELA binding to APJ receptors guides angiogenic cells to migrate toward the midline. In ELA-deficient zebra fish embryos, Chng et al. (6) constructed a homozygous mutant of ELA using zinc finger nuclease technology. The endodermal differentiation potential was impaired, accompanied by decreased expression of GATA binding protein 5 (GATA5) and sex-determining region Y-box (SOX17), suppressed expression of cardiac myosin light chain 2 (Cmlc2), and poor blood circulation. After knockout of the ELA gene in mice, the blood vessels of the offspring showed significant abnormalities, especially characterized by a reduction in the number of blood vessels in the brain, heart, and body segment regions (24, 25). Moreover, the ELA-APJ system activates extracellular regulated protein kinases1/2 (ERK1/2) phosphorylation, inhibits cyclic adenosine monophosphate (cAMP) generation, and regulates calcium ion levels (26). These observations demonstrate that ELA plays an indispensable role in the self-renewal of embryonic stem cells and the development of the cardiovascular system.

### 2.2 The role of ELA in preeclampsia

Preeclampsia (PE) is a multisystem pregnancy disorder characterized primarily by new-onset hypertension after 20 weeks of pregnancy, accompanied by proteinuria, maternal acute kidney injury, and so on (27). Poor placental perfusion leads to trophoblastic cell dysfunction, which affects maternal spiral artery remodeling and ultimately leads to maternal vascular endothelial injury (28). As a pregnancy-related hormone, ELA has been closely linked to the onset of maternal PE. Ho et al. (25) found that ELA knockout affects the formation of placental vasculature, promotes PE in maternal mice, produces proteinuria and elevated blood pressure, and affects fetal angiogenesis, while the administration of exogenous ELA infusion improved the symptoms. Clinical studies have shown that ELA plasma concentrations are elevated in pregnant women with late-onset preeclampsia (LOPE) but not in those with early-onset preeclampsia (EOPE) (29, 30). In contrast, other studies have shown that ELA is lower than normal in PE (31–34), while two other studies reported that plasma ELA level was not related to preeclampsia (35, 36). The ELA detection method, sample selection source, and population selection used in the above studies may all affect the reliability of the results. For example, in the study of Zhou et al., the subjects had a body mass index (BMI) of approximately 25, while Panaitescu et al. enrolled subjects with a BMI of approximately 28. Georgiadou et al. showed that pregnant mothers with higher BMI experience greater cardiovascular stress during pregnancy, which may stimulate the release of ELA into the blood circulation, thereby increasing plasma ELA levels (37). However, another study has shown that ELA is not related to BMI, but to plasma C-reactive protein (CRP) and maternal delivery times (32). In summary, in the current clinical study, ELA changes in PE have yielded inconsistent findings (Table 1), and the results in mouse models do not fully align with those observed in humans. We cannot draw firm conclusions, but the decreasing trends in plasma ELA levels in PE were predominant, and their changing trends and regularity need to be studied with large samples.

**Table 1:** Changes of plasma ELA in PE in clinical studies.

Published time	Disease	Sample number	Changing trends of ELA	Changes in other indicators
February 2020 (29)	EOPE, LOPE	N1 = 56, N2 = 52, EOPE = 54, LOPE = 52	LOPE↑; EOPE, ns	C-fas↑
July 2018 (30)	EOPE, LOPE	N1 = 59, N2 = 60, EOPE = 56, LOPE = 57	LOPE↑; EOPE, ns	/
September 2023 (31)	LOPE	*N* = 60, LOPE = 60	↓	/
May 2023 (32)	PE	*N* = 33, PE = 33	↓	/
May 2019 (33)	PE, SPE	*N* = 30, PE = 28, SPE = 24	↓	NO↓; fetal weight↓
June 2023 (34)	EOPE, LOPE	*N* = 30, EOPE = 30, LOPE = 30	EOPE↓; LOPE, ns	
February 2020 (35)	HDP	*N* = 60, CH = 54, GH = 94, PE = 87, CH/PE = 21	ns	
May 2018 (36)	PE	*N* = 32, PE = 32	ns	C-fas↑

The changing trends of ELA and some other indicators in different types of preeclampsia in previous clinical studies.

“↑” indicates an increase; “↓” indicates a decrease. APJ, apelin receptor; EOPE, early-onset preeclampsia; LOPE, late-onset preeclampsia; HDP, hypertensive disorders of pregnancy; SPE, severe preeclampsia; PE, preeclampsia; CH, chronic hypertension; GH, gestational hypertension; NO, nitric oxide; VEGF-A, vascular endothelial growth factor A; C-fas, markers of preeclampsia; N1, normal; N2, women with normal pregnancies. ns, non-significant difference.

Focusing from clinical phenomena to basic research, it was found that ELA can treat PE through different but largely similar mechanisms of action. Research shows that ferroptosis is involved in the pathogenesis of PE (38). Ferritin heavy chain 1 (FHC1) can resist oxidative stress and maintain normal iron homeostasis, and ELA can increase FTH1 level and inhibit cell ferroptosis (39). Ma et al. (40) showed that ELA can decrease the expression of caspase-3 and Bax and apoptosis of the human choriocarcinoma cell line and increase the junction area of the uterine artery. On the other hand, overexpression of ELA enhances the expression of matrix metalloproteinases, such as matrix metalloproteinase 2 (MMP2) and matrix metalloproteinase 9 (MMP9), via the phosphatidylinositol-3 kinase/protein kinase B (PI3K/Akt) signaling pathway. These enzymes degrade type IV collagen, a key component of the basement membrane of the uterine epithelium, promoting cell invasion and migration, which ultimately facilitates the remodeling of the uterine spiral arteries (41). These results may suggest that ELA could be used as a potential targeted therapy for PE. In addition, the expression of ELA in the human choriocarcinoma cell line and human chorionic cancer cell line was reduced under hypoxic conditions, which may provide further evidence (42). Except for trophoblast cells, the injury of placental vascular endothelial cells is also involved in the pathogenesis of PE. ELA incubation improved cell survival, migration, and tubular formation by upregulating APJ receptors of human umbilical vein cell fusion endothelial cells through activating the PI3K/Akt signaling pathway (43). PE trophoblastic cells are abnormally impaired in endothelial–mesenchymal transition, and high expression of the epithelial marker β-catenin is associated with reduced invasion and migration of trophoblastic cells (44). However, another study found that excessively high concentrations of exogenous ELA can affect the expression of β-catenin through PI3K/Akt, inhibiting the invasion and migration of trophoblast cells (45). In the above clinical study on PE, a significant increase in plasma ELA levels in LOPE was observed. Does this prove that high concentration of ELA has an inhibitory effect, thus leading to the occurrence of PE? Is there an appropriate concentration of ELA in PE, whether higher or lower than this range, that would lead to the occurrence of PE? Of course, elevated ELA levels may also be caused by stress in the maternal cardiovascular system. From the above different studies, ELA can treat PE by reducing oxidative stress, inhibiting ferroptosis, and regulating the invasion ability of trophoblast cells. It plays different or even opposite roles, which may be related to its dose.

## 3 ELA in adulthood: cardiovascular development and diseases

### 3.1 ELA can regulate blood pressure and remodel blood vessels

Clinical research has found that ELA levels gradually decreased as blood pressure rises (46), with the strongest correlation observed with systolic blood pressure (47). ELA-21 can inhibit the expression of inflammatory cytokines, such as tumor necrosis factor-α (TNF-α) and interleukin-6 (IL-6), in spontaneous hypertension rat (SHR) vascular smooth muscle cells and AngII-induced Wistar Kyoto rat control group (48). These inflammatory factors can lead to vascular remodeling and abnormal development. Studies have also demonstrated that ELA may be used as a target to regulate vascular function through the NADPH oxidase/ROS/NLRP3 inflammasome pathway (49). Moreover, it inhibits oxidative stress, inflammation, and apoptosis of aortic outer membrane fibroblasts by acting on fibroblast growth factor 21(FGF21)/angiotensin-converting enzyme 2 in periaortic membrane (ACE2) (50). Through Krüppel-like factor 15 (KLF15)/glutathione peroxidase 4 (GPX4), it downregulates apoptosis- and inflammation-related factors, including interleukin-1 beta (IL-1β) and IL-6. It also upregulates nuclear respiratory factor 2 (Nrf2) and glutathione(GSH) levels (51).

ELA alleviates hypertension and its associated renal dysfunction in hypersalt-fed SHR through anti-inflammatory and antioxidant effects (52). Salt-sensitive rats were injected with ELA after 3 months of a high-salt diet. The blood pressure increased slowly, and the gene expression related to renal fibrosis was reduced (53). Angiotensin I is converted to angiotensin II (Ang II) by angiotensin-converting enzyme (ACE), which acts on the angiotensin II-type 1 receptor (AT1) to promote an increase in blood pressure, left ventricular remodeling, and inflammation. ELA reduces ACE expression, indirectly limiting production of Ang II (54). Intravenous administration of ELA significantly reduces mean arterial pressure. However, injection of ELA into the central nervous system has the opposite effect. Microinjection of ELA-21 into the nucleus paraventricularis increases mean arterial pressure and heart rate in SHR by enhancing sympathetic nerve activity and antidiuretic hormone release through the PI3K/Akt pathway (55). Extensive inflammation and oxidative stress are key events in the occurrence and development of hypertension and cardiovascular complications. Peripherally, ELA can bind to APJ receptors on vascular smooth muscle, inhibit PI3K/Akt, and activate Nrf2, thereby reducing inflammatory responses and the production of reactive oxygen species (ROS) (48, 56). In the central nervous system, continuous infusion of ELA induces sympathetic nerve activation and antidiuretic hormone release, leading to the occurrence of hypertension (55). The mode of action of ELA is a promising direction of future targeting, as its effects may vary depending on its specific mode of action.

In pulmonary arterial hypertension (PAH), the expression of the ELA-APJ axis is significantly decreased in the lung tissue. ELA functions as an endogenous agonist of APJ in the adult cardiovascular system, and exogenous administration can compensate for its downregulation in PAH (57). ELA transfection significantly reduces right ventricular systolic blood pressure and N-terminal pro-brain natriuretic peptide (NT-proBNP), upregulates the expression of pathways such as Krüppel-like factor 2/endothelial nitric oxide synthase (KLF2/eNOS), and inhibits pulmonary arteriole remodeling by weakening endothelial interstitial transformation and media thickening in PAH rats (58).

### 3.2 Can ELA alleviate atherosclerosis?

Currently, the role of ELA in atherosclerosis (AS) is limited. However, it can be confirmed that ELA in systemic circulation of hypertensive patients is significantly reduced and carotid intima–media thickness (IMT, a marker of subclinical AS) is increased (59). In addition, Apelin-13, which plays a similar role and has a high degree of homology with ELA, inhibits the process of AS by impairing Ang II intracellular signaling and increasing nitric oxide (NO) production in apolipoprotein E (APOE)-deficient mice (60). This needs to be further verified by adding an appropriate dose of ELA into the AS model. Atherosclerosis is a complex process involving the combined action of multiple mechanisms, including endothelial cell disorders induced by oxidative stress; infiltration of pro-inflammatory cytokines, chemokines, etc. (61); and phenotypic transformation and migration of vascular smooth muscle cells. ELA has also been proven to be capable of inhibiting the proliferation and migration of vascular smooth muscle cells (55). In addition, external stimuli such as smoking, hyperglycemia, dyslipidemia, or calcification can cause repeated damage to the arterial wall. ELA has the effect of reducing inflammatory infiltration and alleviating oxidative stress (62). The above proves that ELA is highly likely to be involved in the occurrence and development process of AS; more robust research is needed to confirm these associations.

### 3.3 ELA promotes angiogenesis

ELA has the effect of promoting angiogenesis during embryonic development, and in adult individuals, it also seems to play an important role. In cerebral ischemia–reperfusion injury in mice, cerebral blood flow reperfusion is insufficient, and cyclooxygenase oogenesis is stimulated. ELA in combination with APJ activates the yes-associated protein (YAP)/transcriptional coactivator with PDZ-binding motif (TAZ) pathway and improves oxygen–glucose deprivation/reperfusion injury (IR) in mouse brain endothelioma cells. Cerebral blood flow is enhanced, and new functional blood vessels are formed (63). Furthermore, hypoxia-inducible factor and vascular endothelial growth factor are essential substances for important angiogenesis. The activation of both can compensate for the damage caused by ischemia to a certain extent. ELA reduces cardiac injury caused by I/R by activating extracellular regulated protein kinases/hypoxia-inducible factor 1-α/vascular endothelial growth factor (ERK/HIF-1α/VEGF) to promote angiogenesis, inhibiting myocardial apoptosis, mitochondrial dysfunction, and fibrosis through the PI3K/Akt pathway (64). I/R is also one of the causes of acute kidney injury, and the restoration of renal blood supply after ischemia plays an important role in reducing injury. ELA can partially alleviate I/R through APJ to improve renal blood flow (65). Moreover, ELA promotes revascularization after ischemia. In hind limb ischemia ELA knockout mice, vascular endothelial growth factor receptor 2 (VEGFR2) expression was significantly lower than that in ELA WT mice (66). Additionally, after an acute myocardial infarction (AMI), vascular function was impaired, and new blood vessels were restricted. In the AMI mouse, AAV-ELA-32 was injected into the heart from the tail vein, the serum NT-proBNP concentration was decreased at 2 and 4 weeks after surgery, and left ventricular ejection fraction (LVEF) was significantly increased. In the manifestation of microscopic molecules, endothelial proliferation markers CD105, CD31, and von Willebrand factor (vWF) were significantly increased in the ELA-32 treatment group. ELA activates the VEGF/VEGFR2 and jagged canonical notch ligand 1 (Jagged1)/notch receptor 3 (Notch3) pathways through APJ, and new blood vessels are increased (67).

In retinopathy of prematurity (ROP), intraperitoneal injection of ELA can significantly reduce vascularless retinal area and increase blood vessel density, which is still achieved through the ferroptosis pathway. ELA may be a safe and promising strategy for early intervention in ROP (68, 69). Interestingly, our team also found that ELA promotes angiogenesis in diabetic foot ulcers. It can alleviate ROS-induced DNA damage and promote endothelial cell migration and angiogenesis by inhibiting tumor necrosis factor receptor 1 (TRAF1) downregulation of nuclear factor kappa-B (NF-κB) signaling (70). To sum up, ELA may have a certain promoting effect on angiogenesis.

### 3.4 ELA can reduce vascular calcification

Vascular calcification is an important pathophysiological process related to age-related CVDs (71). Abnormal deposition of calcium and phosphorus minerals on the vessel walls leads to vessel hardening and reduced elasticity. ELA improves age-related vascular calcification by preventing cuproptosis, maintaining mitochondrial membrane potential, and reducing mitochondrial ROS production. Interestingly, this study additionally found that ELA mitigated cell aging and the production of pro-inflammatory cytokines (72). This also suggests that ELA may be an effective treatment for the management of delayed aging and age-related diseases.

### 3.5 The role of ELA in heart disease

ELA may be a promising therapeutic target for heart diseases (Table 2). In heart diseases, ELA has different changing trends (73–81). Notably, when ELA levels exceeded 9.5 ng/ml, the diagnostic sensitivity and specificity of complete atrioventricular block were 90.2% and 88.0%, respectively (82). It can be seen that the increase or decrease of ELA levels in a series of heart diseases is uncertain and may be related to the severity of the disease. To compensate for the harm caused by it, ELA levels increase. A relevant study has shown that an increased endogenous ELA level is correlated with the degree of coronary artery stenosis and is positively correlated with coronary artery disease within a certain range; however, it is not significantly correlated with the degree of coronary artery disease beyond or below this range (63.47–85.49 ng/ml) (83). It is also worth noting that Apelin has been reported to promote platelet aggregation through signaling pathways (84). Given that ELA plays a similar biological role to Apelin, will it aggravate the occurrence of diseases such as thrombosis or coronary artery occlusion? However, how it changes in these disease states needs more research.

**Table 2:** Changes of plasma ELA in CVDs in clinical studies.

Published time	Disease	Sample number	Changes of ELA	Relationship between relevant indicators and ELA
February 2024 (47)	Hypertension	MHT = 25, BHT = 25, *N* = 25	↓	BMI, SBP, DBP, UA, BUN, Scr↓; eGFR↑
May 2020 (46)	Hypertension	EH = 31, *N* = 31	↓	DBP↓; FMD↑
October 2020 (82)	AV	AV = 50, *N* = 50	↑	NT-proBNP↑; HR↓
Novermber 2020 (76)	RVOTO	PS/PA/IVS = 51; *N* = 16	↓	/
September 2021 (77)	CTO	CTO = 50, *N* = 50	↓	NYHA, LVEF, LVEDV, mortality, hospitalization rate↑
October 2023 (78)	HFrEF	HFrEF = 73, *N* = 77	↑	/
Published time	Disease	Sample number	Changes of ELA	Relationship between relevant indicators and ELA
July 2021 (80)	CHD, (SA/UAP/AMI)	SA = 59, UAP = 38, AMI = 41, *N* = 86	UAP, AMI↑	Hs-troponin T, CK-MB↑
September 2021 (81)	CCS, ACS	CCS = 30, ACS = 84, *N* = 33	ACS↑	BNP, LVDD, LVEDD, LVPW↓; LVEF↑
May 2024 (73)	HF	HF = 216, *N* = 119	↓	/
December 2021 (74)	AF	AF = 103, PSVT = 28	↓	BNP↓
August 2021 (75)	Hypertension	HT = 81, HT/AF = 81	↓	/
October 2019 (79)	STEMI	STEMI = 124, *N* = 77	↑	LVEF↓; troponin T, NT-proBNP↑

The changing trends of ELA and some other cardiovascular function indicators in different cardiovascular diseases in previous clinical studies.

“↑” indicates an increase; “↓” indicates a decrease. AAF, atrial fibrillation; ACS, acute coronary syndrome; AV, complete AV block; ABI, ankle brachial index; BHT, benign hypertension; BNP, B-type natriuretic peptide; BMI, body mass index; BUN, blood urea nitrogen; CCS, chronic coronary syndromes; CHD, coronary heart disease; CTO, chronic total occlusion; CK-MB, creatine kinase-myocardial band; DBP, diastolic blood pressure; EH, essential hypertension; eGFR, estimated glomerular filtration rate; FMD, flow-mediated dilation; LVEF, left ventricular ejection fraction; GH, growth hormone; LEAD, lower extremity artery disease; IVS, intact ventricular septum; Hs-CRP, high-sensitivity C-reactive protein; Hs-troponin T, high-sensitivity troponin T; HR, heart rate; LVDD, left ventricular end diastolic dimension; HFrEF, heart failure with reduced ejection fraction; Hs-CRP, hypersensitive C-reactive protein; LVEDV, left ventricular end diastolic volume; LVEDD, left ventricular end diastolic diameter; LVPW, left ventricular posterior wall dimensions; MHT, malignant hypertension; PAD, peripheral arterial disease; PA, pulmonary atresia; PS, pulmonary stenosis; NT-proBNP, N-terminal pro-B-type natriuretic peptide; PSVT, paroxysmal supravent ricular tachycardia; SA, stable angina; Scr, serum creatinine, STEMI, ST-segment elevation SBP, systolic blood pressure; myocardial infarction; T, troponin; UA, unstable angina; UAP, unstable angina pectoris; RVOTO, right ventricular outflow tract obstruction.

In basic research, ELA was found to be significantly elevated in postinfarction cardiac remodeling, thereby promoting increased cardiac contractility through activation of ERK1/2 signaling, which may mitigate the adverse consequences of myocardial infarction (MI). This finding was also consistent with the expression of ELA in the serum of clinical MI patients (85). Exogenous ELA can significantly reverse Ang II-mediated pathological myocardial remodeling, dysfunction, and ultrastructural injury. After ELA knockdown, cell proliferation and migration of cardiac fibroblasts are significantly enhanced, which is accomplished by regulating the ferroptosis pathway (62). In Dox-induced acute cardiac injury, ELA alleviates myocardial necrosis and restores autophagy in neonatal rats by upregulating the T-cell transcription factor EB (86). In diabetic cardiomyopathy, it inhibits oxidative stress and inflammation through sirtuin3 (SIRT3)-mediated forkhead box O3 (FOXO3) deacetylation (87). Subcutaneous injection of 1 mg/kg/ day of ELA for 2 weeks was found to inhibit myocardial autophagy and cellular oxidative stress, which was also observed in LPS-modeling mice with septic myocardial injury (88). All these may have laid a further foundation for the treatment of ELA in CVDs and highlight ELA’s protective potential.

### 3.6 The role of ELA in arterial disease

Clinical studies have shown a significant inverse correlation between critical carotid artery disease and ELA levels in patients with non-cardioembolic ischemic stroke (89). In addition, the expression of ELA has been found to increase in patients with lower extremity arterial disease (90). There are relatively few clinical studies on this aspect, and more clinical patients are needed.

**Figure 2:**
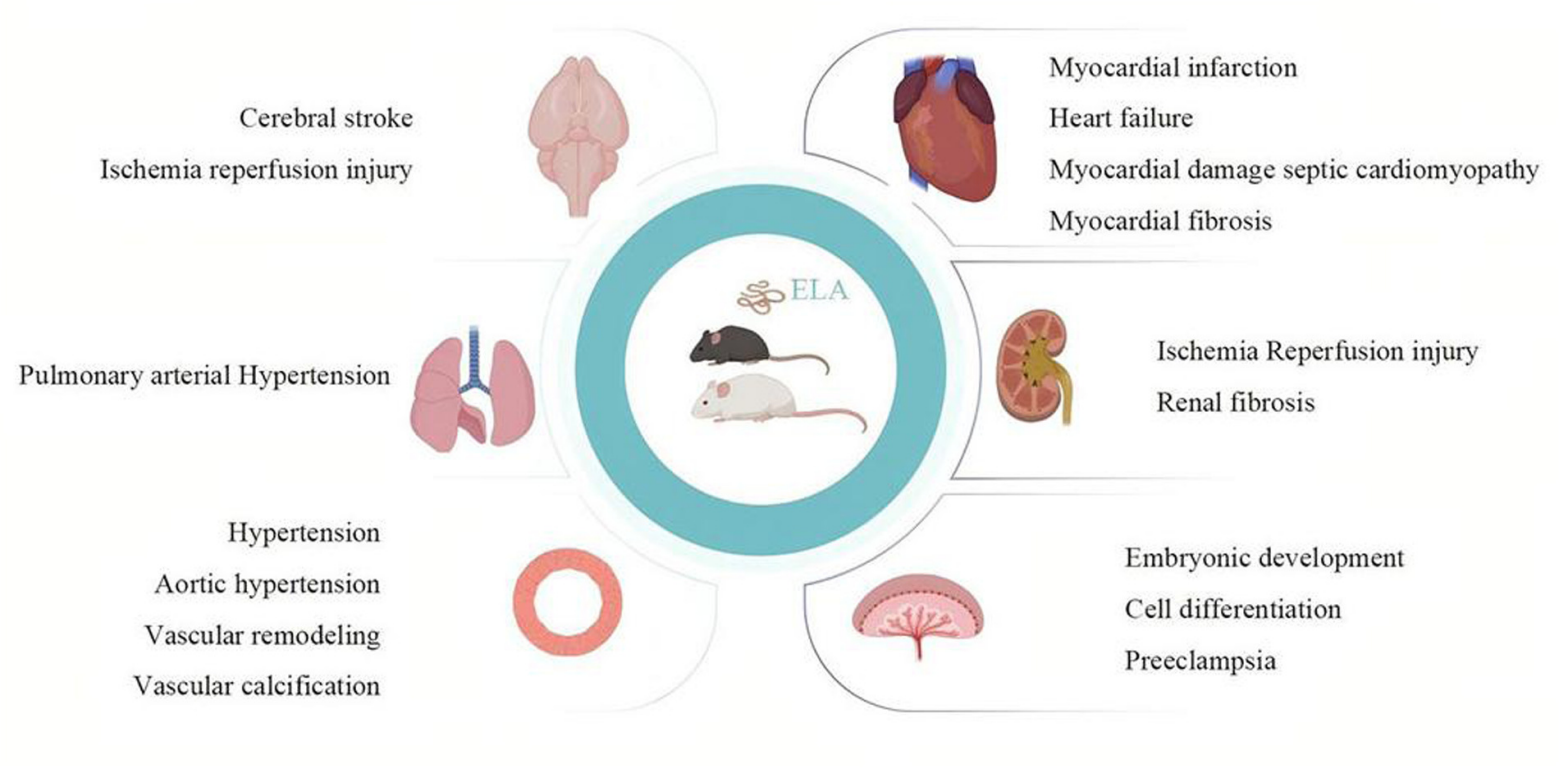
Pathophysiological role of ELA in the cardiovascular system. The figure shows the therapeutic effect of ELA on cardiovascular development and related diseases in basic experimental research. The main organs or tissues it acts on are the heart, brain, lungs, kidneys, blood vessels, and placenta. The main disease patterns include myocardial infarction, heart failure, septic cardiomyopathy, myocardial fibrosis, stroke, ischemia–reperfusion injury, pulmonary hypertension, hypertension, aortic hypertension, vascular remodeling, vascular calcification, preeclampsia, embryonic stem cell differentiation, and embryonic development. Created in Biorender.

**Figure 3:**
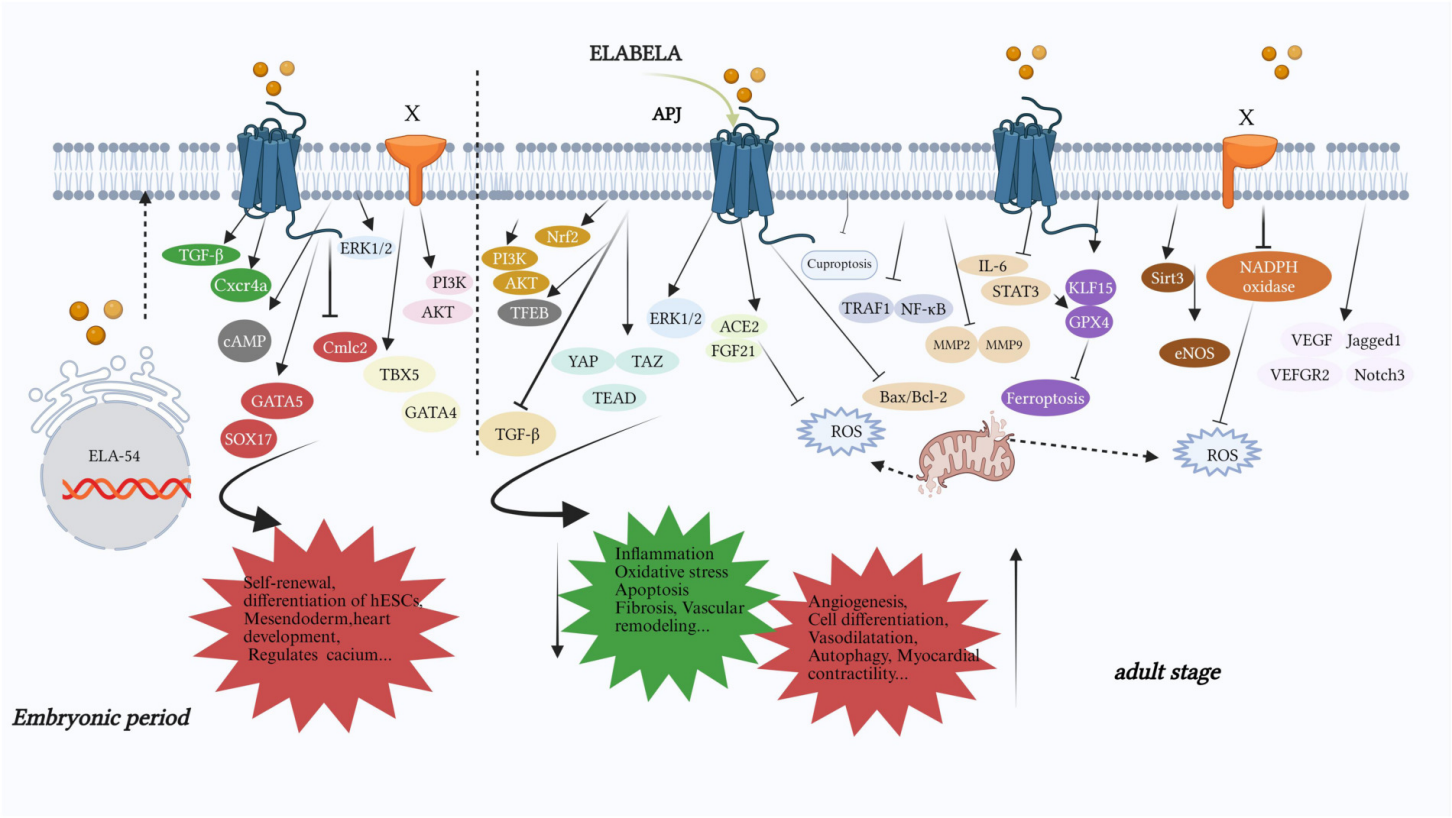
The part of the signaling pathway or important molecules in which ELA mainly plays a role. During the embryonic period, it mainly promotes cardiac development (GATA5, SOX17, Cmlc2), self-renewal (PI3K/AKT) and differentiation (TBX5, GATA4) of embryonic stem cells, regulation of calcium ion levels (cAMP), and mesendoderm development (TGF-β, Cxcr4a). In adulthood, ELA mainly reduces oxidative stress (PI3K/AKT, Nrf2, autophagy, ROS, KLF15/GPX4, TRAF1/NF-κB, IL-6/STAT3/GPX4, Sirt3, NADPH oxidase, cuproptosis), anti-inflammatory effect (FGF21/ACE2, TRAF1/NF-κB, MMP2/9, Sirt3, NADPH oxidase, cuproptosis), apoptosis (PI3K/AKT, ERK1/2, FGF21/ACE2, Bax/Bcl2, Sirt3), organ fibrosis (PI3K/AKT, ACE2/GDF15, Sirt3), vascular remodeling (IL-6/STAT3/GPX4, eNOS, NADPH oxidase) and promotes autophagy (TFEB), angiogenesis (PI3K/AKT, YAP/TAZ/TEAD, ERK, IL-6/STAT3/GPX4), the recovery of cardiovascular functionand, and so on. X: an unknown receptor for ELA; the PI3K/AKT pathway in the embryonic period and the NADPH pathway in adulthood function through unknown receptors, while other signaling pathways function through APJ. TBX5, T-box transcription factor 5; GATA4, GATA binding protein 4; GATA5, GATA binding protein 5; Sox17, sex-determining region Y-box 17; Cmlc2, cardiac myosin light chain 2; Cxcr4a, chemokine (C-X-C motif) receptor 4a; Nrf2, nuclear factor erythroid 2-related factor 2; TFEB, transcription factor EB; ACE2, angiotensin-converting enzyme 2; YAP/TAZ/TEAD, yes-associated protein/tTranscriptional coactivator with PDZ-binding motif/transcriptional enhanced associate domain; STAT3, signal transducer and activator of transcription 3; GPX4, glutathione peroxidase 4; KLF15, Krüppel-like factor 15; eNOS, endothelial nitric oxide synthase; TRAF1, tumor necrosis factor receptor-related factor 1; Jagged1, jagged canonical notch ligand 1; Notch3, notch receptor 3; FGF21, fibroblast growth factor 21.

From the above, ELA may play a significant role both during the embryonic period and in adulthood. Animal research indicates that its main organizing organs include the heart, brain, lungs, kidneys, placenta, and blood vessels (Figure 2). As of the current research, ELA can alleviate oxidative stress and reduce fibrosis and damage through different pathways (Figure 3). These findings support the potential of ELA as a target for the diagnosis and treatment of CVDs.

## 4 Conclusion and prospect

From basic research to clinical application, there are still several key challenges, such as stability issues and short half-life. ELA is a small peptide and is easily degraded by DPP-4 and neutral endopeptidases in plasma. Some studies have fused the IgG-Fc-ELA protein, which can extend its half-life to 44 h. Furthermore, so far, the main known targets of ELA are still APJ. Developing peptide agonists or inhibitors that act on APJ is another approach, such as ML221 and CMF-019. In addition, targeted delivery strategies may involve developing tissue-specific delivery systems, such as nanoparticles and exosomes, which play an important role in the application of peptide drugs, especially in cerebral ischemia–reperfusion injury, where they can cross the blood–brain barrier. It should also be noted that the current research on ELA and its main receptor APJ has only included basic animal observations and preliminary clinical observations. Further verification in primates or humans is needed to clarify the characteristics, mechanisms of action, oral effects, plasma bioavailability, and stability of this molecule. There is a long way to go. The potential of ELA is limited by clinical transformation obstacles, but we also believe that in the future, it can truly serve clinical practice.
